# Pattern of Consumption of Sports Supplements of Spanish Handball Players: Differences According to Gender and Competitive Level

**DOI:** 10.3390/nu16020315

**Published:** 2024-01-20

**Authors:** David Romero-García, José Miguel Martínez-Sanz, Jaime Sebastiá-Rico, Carmen Manchado, Raquel Vaquero-Cristóbal

**Affiliations:** 1 Nursing Department, Faculty of Health Sciences, University of Alicante, 03690 Alicante, Spain; drg33@gcloud.ua.es; 2 Area of Nutrition, University Clinic of Nutrition, Physical Activity and Physiotherapy (CUNAFF), Lluís Alcanyís Foundation—Universiy of Valencia, 46020 Valencia, Spain; jaime.sebastia@fundacions.uv.es; 3 Food & Health Lab, Institute of Materials Science, University of Valencia, 46980 Valencia, Spain; 4 General Didactics and Specific Didactics, Faculty of Education, University of Alicante, 03690 Alicante, Spain; carmen.manchado@ua.es; 5 Department of Physical Activity and Sport Sciences, Faculty of Sport Sciences, University of Murcia, 30720 Murcia, Spain; raquel.vaquero@um.es

**Keywords:** sports supplements, handball, sport nutrition, sport performance

## Abstract

(1) Background: Given the physiological characteristics of handball, players may require the use of certain sports supplements (SS). However, very few studies have investigated the consumption of SS in handball. The aims were to determine the number of handball players who consume SS, to analyze their SS consumption pattern according to gender and competitive level, and to assess whether the SS they consume are supported by scientific evidence, and to which group they belong according to the classification of the Australian Institute of Sport (AIS). (2) Methods: A descriptive-correlational study was carried out on the habitual consumption of SS in 360 federated Spanish players by using a self-administered and validated questionnaire. (3) Results: These showed 65.8% of the sample consumed SS. According to the total number of participants, the most consumed supplements were from Group A: sports drinks (30.8%) and whey protein (30.4%). When analyzing the data by gender, the men’s consumption was significantly higher for whey protein (*p* < 0.001), caffeine (*p* = 0.009), and creatine monohydrate (*p* < 0.001). When analyzed by competitive level, the provincial category players group showed a significantly lower consumption than the rest of the groups for protein bars (*p* = 0.038), whey protein (*p* = 0.005) and creatine monohydrate (*p* < 0.001), while the honor division group showed a significantly higher intake of creatine monohydrate than the remaining groups (*p* < 0.001). (4) Conclusions: The handball players showed a moderate consumption of SS, without using substances that were not supported by scientific evidence and opting in most cases for supplements belonging to group A from the AIS classification. Men tended to consume more SS, and SS consumption increased based on competitive level.

## 1. Introduction

Handball is a team sport that is characterized by interspersing high-intensity actions, such as sprints, throws, or changes in direction, with brief moments of low intensity that serve as recovery periods between these explosive actions [1,2,3]. It is a complex, multifactorial and very demanding sport, in which players need to have an optimal physical preparation to achieve maximum performance in all of these actions [4,5].

One of the aspects that most influences performance improvement is the dietary–nutritional planning of the athlete, since it is responsible for ensuring the necessary food intakes to cover all the energy–nutritional requirements needed during sports practice and sports performance [6,7]. A key part of this planning is the use of sports supplements (SS), which can be utilized to directly improve performance during matches, to improve recovery between training sessions, or simply to prevent and reduce injuries and illnesses [8]. However, it should be kept in mind that the basis of an athlete’s nutrition should be a balanced diet, and supplements are only a complement that are not necessary for everyone [7].

In the latest consensus statement from the International Olympic Committee [9], a SS is defined as a food, food component, nutrient, or non-food compound that is purposefully ingested in addition to the customary diet consumed for the purpose of achieving a specific health and/or performance benefit. SS can be found in many forms. In addition, SS are usually categorized according to the classification proposed by the Australian Institute of Sport (AIS) [10], according to the level of scientific evidence and practical applications that determine their safety, legality and effectiveness in improving sports performance. This classification is divided into four groups (A, B, C and D) and its latest updated version was in 2022. Group A SS are those with a strong level of scientific evidence for use during exercise, using evidence-based protocols. Group B SS are those that are still being studied and therefore require further research, and should be used with caution. Group C includes SS whose benefits have not been supported by scientific evidence or have not been sufficiently researched, so their consumption is not recommended. Finally, Group D SS are banned or at high risk of contamination with substances that could lead to a positive doping test, so athletes should not consume them. With the current boom in SS consumption, this classification serves the technical–medical staff around the players as a guide on which supplements to advise, intake protocol, adverse effects, as well as to avoid possible cases of doping [10].

Numerous studies have been found on the consumption of SS in endurance sports such as open water swimming [11], mountain running [12] or triathlon [13], showing a SS consumption of 79.5% in swimmers, 93.8% and 87.5% in runners, and 92.2% in triathletes. This is probably because endurance and ultra-endurance athletes often train and compete in a self-sufficiency or semi-sufficiency regime, forcing them to carry their own food and hydration, so that the SS help them to optimize the space and weight of their sports equipment [11,12,13]. Less research has been conducted on team sports. However, a study evaluating 912 professional athletes in four team sports (basketball, volleyball, handball and soccer) showed that the percentage of players consuming at least one SS was reduced by almost half (52.6% in basketball, 46.3% in soccer, 46.8% in volleyball and 48.5% in handball) as compared to previous data from endurance sports [14]. However, this study did not consider the gender of the athletes or the competitive level, in addition to not assessing the scientific evidence of the supplements consumed using the AIS classification. More recent studies on other team sports found, on the one hand, that 65.3% of rugby players consumed SS, showing a higher prevalence in men than in women, and in professional versus amateur players [15]. On the other hand, in soccer, they found that 84.1% of Spanish elite female soccer players consumed SS [16] and 87.2% of Turkish soccer players consumed SS, finding a higher prevalence in men and professionals, as compared to women and non-professionals, respectively [17]. Not surprisingly, only one study has specifically analyzed the consumption of SS in handball players [8], finding that 59.9% of handball players consumed SS, with no significant differences between genders or between professional and amateur handball, and with a relatively low use of those supplements with a low level of scientific evidence. However, in this study, the classification of the competitive level between professional and amateur could have certain limitations, as it only considered first division players as professionals, when second division players could also be considered as such. Therefore, the aims of the present study were: (a) to determine the number of handball players who consume SS; (b) to analyze their consumption pattern, according to gender and competitive level; and (c) to assess whether the SS they consume are supported by scientific evidence and to which group they belong according to the AIS classification.

As for the main hypotheses of this work, the following are proposed:

**Hypothesis** **1** **(H1).** *The number of handball players who use sports supplements will be higher as compared to those who do not use them.*

**Hypothesis** **2** **(H2).** *No differences are expected between the total consumption of sports supplements between men and women, but differences are expected between levels of competition, being higher as the competitive level increases.*

**Hypothesis** **3** **(H3).** *The handball players will correctly choose the supplements, with most of them belonging to Group A of the AIS classification.*

## 2. Materials and Methods

### 2.1. Type of Study

Descriptive cross-sectional study on the consumption and habitual use of sports supplements by handball players in Spanish leagues. The sample size was calculated using Rstudio software (version 3.15.0, Rstudio Inc., Boston, MA, USA). The significance level was set a priori at *p* = 0.05. The standard deviation (SD) was set according to total SS intake data from previous studies on Spanish elite athletes (SD = 2.1) [18]. With an estimated error (d) of 0.21, the required sample size was 360 subjects. The study population was selected by non-probability convenience sampling from handball federations and clubs throughout Spain. The protocol followed the World Medical Association codes and the Declaration of Helsinki for research in humans at all times and was approved by the ethics committee of the University of Alicante, with file number UA-2022-02-01. In addition, the study design, as well as the development of the manuscript, followed the STROBE statement [19].

### 2.2. Participants

A total of 360 handball players voluntarily participated in the study. They had a mean age of 23.13 ± 5.36 years and an age range from 18 to 35 years old. The sporting experience according to the participants’ years in the federation was 8.70 ± 2.20 years. Regarding weekly training time, 29.2% trained between 1 h and 1.5 h, 48.6% trained between 1.5 h and 2 h and 22.2% trained for more than 2 h. Of the total, 199 were men and 161 were women, all of whom were of legal age. The participants were also divided by level of competition into provincial (competitions held in each province and community), national (competitions held throughout Spain) and honor division (competitions that are also held at the national level and the top teams can even play in international competitions). The inclusion criteria for the study were: (a) to be of legal age; (b) to have been playing handball for at least two years; (c) to be federated in handball; (d) to play in one of the levels of competition described; and (e) to complete the survey in its entirety. The exclusion criteria for the study were: (a) being injured at the time of the study; (b) having had an injury in the three months prior to the study. Table 1 describes the players’ data regarding age, basic anthropometric characteristics and training days of the participants, segmented by gender and level of competition, as well as the Student’s *t*-test and ANOVA, respectively.

### 2.3. Procedure

First, a list was made of the contact information of the national federation, autonomous handball federations of Spain, and clubs, with the data reported on the web pages of the National Handball Federation and the territorial federations. The representatives from the national federation and each autonomous handball federation in Spain, as well as the handball clubs registered in these federations, were contacted by email. They were informed of the characteristics of the study and their collaboration was requested. Once they agreed to participate, they were sent an email with a link to the supplement consumption questionnaire, which the handball players could complete voluntarily, electronically and anonymously. The questionnaire was active during the 2022–2023 competitive season, from February to May 2023.

### 2.4. Instruments

A questionnaire that had been previously used in similar studies was distributed [8,11,12,13,20]. The selected supplement consumption questionnaire, whose author is Sánchez-Oliver, was validated for content, applicability, structure and presentation [21]. This questionnaire was created by a team composed of three scientists specialized in sports, and a total of 25 experts in various areas such as sports science, sports medicine, nutrition, chemistry and pharmacology, who were in charge of verifying its construction validity [21]. In fact, in a study conducted by Knapik et al. [22], who analyzed the quality of questionnaires designed to determine the prevalence of SS use by athletes, this form obtained a methodological score of 54%. It stood out as one of the 57 questionnaires reviewed (out of a total of 164) considered adequate to collect accurate information on SS used by athletes. The questionnaire contains a total of 34 questions divided into three main sections. The first section, consisting of 6 questions, collects anthropometric data such as age, body mass, height; personal data such as gender and date of birth; and social data such as autonomous community of residence. The second section includes 10 questions covering the practice of the sport and its context, the club in which it is played, the league in which competitions take place, the years of practice, and the training and match schedules. The last section, consisting of 14 questions, focuses on the consumption of supplements, in other words, what supplements they consume, why they consume them, who advises them to consume them, where they buy them, when they take them, and the perception they have after consuming them. In this last part, there are both dichotomous questions (yes or no) about whether or not they are consuming SS at the time they complete the questionnaire, and a list of supplements where players mark the supplements they are consuming, making a final count of how many supplements they consume. The total consumption of each of the groups is also obtained, considering how each supplement is classified according to the AIS classification. The questionnaire can be found as Supplementary Material File S1.

### 2.5. Statistical Analysis

The Kolmogorov–Smirnov test was applied to assess whether the variables had a normal distribution, and the Levene test to verify homoscedasticity. Given their normal distribution and with the objective of describing the sample, the mean was used together with its standard deviation (mean ± SD). To analyze the differences in the consumption of SS from the different categories determined by the AIS, for gender (male–female) a Student’s *t* test was performed, and the effect size was calculated using Cohen’s d coefficient, and for the level of competition (provincial, national and honor division), an ANOVA analysis was performed and the effect size was calculated with the eta squared measure (η^2^). The significance level was established a priori at *p* < 0.05. For the segmentation of the level of competition, a pairwise comparison was performed after Bonferroni adjustment (*p* < 0.016) of the variables measured in which significant differences were found. On the other hand, both for SS consumption and for those supplements that were consumed by more than 10% of the sample, a chi-square test (X^2^) was performed to verify possible differences according to gender and level of competition. Residuals and Cramer’s V were also calculated to analyze the association between the different groups. The significance level was set at *p* < 0.05. The statistical analysis was performed with the program Statistical Package for Social Sciences (SPSS) v.25 (IBM, Armonk, NY, USA).

## 3. Results

### 3.1. Overall Consumption of Sports Supplements, According to Gender and Level of Competition

Of the total sample, 65.8% of the handball players reported consuming sports supplements. On the one hand, with respect to gender, it was observed that 61.5% of the women and 69.3% of the men consumed SS, with no significant differences between them (*p* = 0.118). On the other hand, with respect to the level of competition, it was observed that 49.0% of the provincial level, 67.9% of the national level and 80.9% of the honor division level consumed SS, and in this case significant differences were found (*p* < 0.001).

Table 2 shows the descriptive data, Student’s *t* test and ANOVA of the sports supplements consumed in the segmented sample according to gender and level of competition. Regarding gender, significant differences were found in the consumption of medical supplements (t = 2.240; *p* = 0.024) and performance supplements (t = 4.638; *p* < 0.001), both cases belonging to Group A supplements, and significant differences were also found for the total supplements from Group A (t = −2.226; *p* = 0.027). For the total Group A supplements and performance supplements, it was observed that the men’s consumption was higher, while women consumed more medical supplements. However, no significant differences were found for sports foods belonging to Group A supplements (t = −1.811; *p* = 0.071), for Group B supplements (t = 0.716; *p* = 0.474) or for Group C supplements (t = 0.482; *p* = 0.630). Regarding competition levels, significant differences were found for total SS (F = 7.869; *p* < 0.001), for all variables in Group A (F = 4.591–7.234; *p* = 0.011–0.001), with the exception of medical supplement consumption (F = 1.132; *p* = 0.324), and for supplements in Group B (F = 4.101; *p* = 0.017) and Group C (F = 4.125; *p* = 0.017). It was also observed that no participants consumed Group D supplements.

Table 3 shows the pairwise comparison between the different categories after the Bonferroni adjustment of the variables measured with significant differences, depending on the level of competition. For total sports supplements, total Group A supplements, and sports food belonging to Group A supplements, significant differences were found when comparing the provincial level with the rest of the levels (*p* = 0.001–0.017). In all cases, the consumption of the national level and the honor division level athletes was higher than the consumption of the provincial level ones. On the other hand, for the performance supplements belonging to Group A supplements, Group B supplements and Group C supplements, significant differences were only found when comparing the provincial level with the honor division level (*p* = 0.013–0.014), and in all cases, the consumption of players at the honor division level was higher.

### 3.2. Most Consumed Sports Supplements, According to Gender and Competitive Level

Table 4 shows the supplements that were consumed by more than 10% of the total sample and according to gender and level of competition. It was observed that sports drinks (30.8%) and whey protein (30.4%) were the most consumed supplements by the total sample. Regarding gender, significant differences were found in the consumption of whey protein (*p* < 0.001), caffeine (*p* = 0.009) and creatine monohydrate (*p* < 0.001), with a significantly higher consumption by men in all three cases. Regarding the level of competition, significant differences were found for protein bars (*p* = 0.038), whey protein (*p* = 0.005) and creatine monohydrate (*p* < 0.001). For protein bars (*p* = 0.038), whey protein (*p* = 0.005) and creatine monohydrate (*p* < 0.001), the provincial category players group showed a significantly lower consumption than the rest of the groups. On the other hand, the honor division group showed a significantly higher intake of creatine monohydrate (*p* < 0.001) than the rest of the groups.

## 4. Discussion

The first aim of the study was to determine the number of handball players who consume SS. In our results, 65.8% of the players reported to be consuming SS at the time of the study, with no significant differences between genders, although significant differences were found between competitive levels. Along this line, there is little scientific literature addressing SS consumption habits in handball. However, a scientific article similar to present one was previously published, which analyzed the use of SS in 187 handball players by examining consumption patterns according to gender and competitive level, where 59.9% of players reported using at least one supplement, with no significant differences between men and women or between professional and amateur players [8]. Another previously published study by Sekulic et al. [14] analyzed the SS consumption of 206 professional handball players, along with other Olympic team sports, and observed that 48.5% consumed SS; however, among this percentage, 33.0% of the players consumed SS rarely or from time to time and only 15.5% of the players took them regularly. It should be noted that the use of SS in team sports is lower than in other sports, such as endurance sports, where the prevalence of use is more than 85% [11,12,13]. It is possible that this increase in SS consumption in our research may be due, on the one hand, to the larger sample size or, on the other hand, to the booming SS market due to increased investment by supplement companies [6,7,9], so research is still lacking in this regard. In summary, the first hypothesis can be accepted because there are more players who consume SS as compared to those who do not consume SS.

Another aim of this research was to analyze the pattern of SS consumption in handball players, both in general and according to gender and competitive level. With respect to the total consumption by handball players in general, in the previously published study, the most consumed SS were sports drinks (42.2%), sports bars (35.3%) and caffeine-containing products (31.6%), with all of these SS categorized by the AIS in group A of scientific evidence [8,10]. Meanwhile, in our study, sports drinks (30.8%), sports bars (27.0%) and caffeine (28.6%) were also widely consumed, but whey protein (30.4%) and creatine monohydrate (29.2%) also stood out. These differences could be explained by the fact that our study included a larger sample of athletes (360 handball players as compared to 187 in the study by Muñoz et al.), so there was a greater probability of observing a greater variety in SS consumption habits [8].

Regarding performance supplements, caffeine and creatine monohydrate stood out as being widely used and researched in team sports, including handball [8,23,24,25,26,27,28,29,30]. Caffeine is the most widely used psychoactive substance in the world, recognized by the International Olympic Committee for effectively improving performance in various sports disciplines [9]. Numerous studies suggest an increase in performance following ingestion of 3–6 mg·kg^−1^ consumed ~60 min prior to exercise [9,30,31,32]. It is suggested that caffeine exerts its influence on the central nervous system by antagonizing adenosine receptors, resulting in increased neurotransmitter release, increased motor unit firing rates and pain suppression [32]. A study in female handball players observed that caffeine supplementation at a dose of 6 mg·kg^−1^ improved cognitive and physical performance, suggesting that female handball players with intermediate circadian phenotypes, that is, those players who focus their mental and physical resources during the mid-morning or early afternoon, might consider caffeine administration prior to morning competition [24]. However, the ergogenic effect of caffeine could be influenced by genetic variations in the CYP1A2 and ADORA2A genes of athletes, as demonstrated in a study conducted in 31 professional handball players with a dose of 3 mg·kg^−1^ [25]. Therefore, personalized ergogenic strategies could be designed in the future based on the genetics of the athletes.

Creatine is a non-protein amino acid, produced in the body from arginine, glycine and methionine in the kidneys and liver [33]. It can also be obtained through the intake of meat or as a dietary supplement [33,34]. However, various forms of creatine supplementation exist, including creatine salts, creatine complexed with other nutrients, or creatine dipeptides, but the scientific literature seems to indicate that the chemical form creatine monohydrate is the most supported by scientific evidence for physiological effects on intramuscular creatine stores and/or performance [33,34,35]. The recommended dosage of creatine to achieve muscle saturation levels and ultimately enhance performance is 3–5 g/day or 0.1 g·kg of body mass/day for at least 4 weeks [33]. Creatine increases intramuscular creatine levels, leading to increased availability of phosphocreatine (PCr), which is used to produce adenosine triphosphate (ATP) during high-intensity exercise [33,34]. Therefore, increasing muscle creatine and PCr stores may improve training adaptations and high-intensity exercise capacity in sports such as handball [26,27,28,29,34]. Several studies have examined the efficacy of creatine in team sports including soccer [36,37], basketball [38,39] and handball [26,27,28,29]. A study with 14 female handball players showed that creatine supplementation for 12 weeks at ergogenic doses improved physical performance and body composition, but the timing of supplementation did not appear to have a significant effect [28]. Other studies have found that elite handball players can experience improved sports performance and recovery through the ingestion of high doses of creatine in a short time frame. These dosages were as follows: 15 g/day for 5 days [27], 20 g/day for 5 days [26], and 60 g/day for 3 days [26].

Medical supplements, including vitamin D and multivitamins, were also consumed, which are supplements used for the purpose of preventing or treating clinical problems, encompassing diagnosed nutritional deficiencies. It is imperative to integrate them as part of a more comprehensive approach, under the direction of a medical professional or an appropriately credentialed sports dietitian [10]. On the one hand, minerals like magnesium have been shown to play a crucial role in muscle function, energy production and recovery after exercise. In addition, magnesium deficiency can negatively affect athletic performance and increase the risk of injury [39,40]. Furthermore, positive results of magnesium supplementation in handball players have been observed, where a significant increase in magnesium concentrations in erythrocytes of the players was confirmed [41]. On the other hand, adequate vitamin intake plays a critical role in sports performance, as these molecules act as cofactors in numerous metabolic reactions that regulate energy production, immune function and tissue repair, and are essential for maintaining physiological homeostasis and optimizing adaptation to training in high-performance athletes [42]. A study conducted on elite handball athletes suggested that they have a different gene expression profile of key genes related to sports performance as compared to sedentary individuals. In addition, multivitamin supplementation had a positive impact on gene regulation in athletes [43]. Periodic monitoring of micronutrients is crucial, particularly for vitamin D and iron metabolism, as they are closely linked to health and athletic performance. This will enable the establishment of appropriate dietary and nutritional recommendations, as well as food education interventions in cases of deficiency or risk of deficiency [6,10].

Sports foods were one of the SS groups most frequently consumed by handball players, with sports drinks (30.8%) and whey protein (30.4%) standing out as the most frequently consumed foods, followed by sports bars (27.0%) and protein bars (25.6%). Sports drinks and sports bars provide rapidly absorbing carbohydrates such as glucose or maltodextrin along with slowly absorbing carbohydrates such as fructose to restore the muscle and hepatic glycogen depleted during physical activity [10]. They also contain minerals to replenish electrolytes lost through sweat. Furthermore, certain ergogenic substances, including caffeine and amino acids such as taurine, may enhance athletic performance. Numerous scientific studies have demonstrated a positive association between carbohydrate consumption derived from food and sports supplements, such as sports drinks and bars, and improved performance in team sports such as soccer [31,44,45]. Furthermore, the consumption of this type of sports supplement can help handball players meet their carbohydrate (30 g/h) and fluid (400–800 mL/h) intake requirements during training and competitions lasting over an hour [6,7]. Additionally, consuming carbohydrates is crucial for replenishing glycogen stores, meeting the energy requirements of the immune system, and repairing damaged tissues post-workout [46]. Therefore, the high consumption of these supplements in our sample of handball players is reasonable.

Protein intake contributes to the synthesis of muscle mass and the maintenance of bone health, as well as increased strength, improved post-exercise recovery, enhanced immune system response and reduced likelihood of musculoskeletal injury [30,47,48]. Although dietary protein intake may be sufficient [48], the amount of food needed to meet the requirements of a protein-rich diet must be considered. Furthermore, there may be limited time available to recover between training sessions and matches [47,49]. Whey protein and protein bars are practical and safe supplements for optimizing the intake of adequate quantities and quality of protein, while also providing good digestibility and helping one adhere to a high-protein diet [10]. It is reasonable to assume that our sample of handball players follows the frequent consumption of these substances observed in previous research on team sports [8,23,31,50,51]. The consumption of protein and carbohydrate supplements, such as powder or bars, can help meet the post-exercise recommendations for these nutrients, which are approximately 1 g/kg for carbohydrates and 0.2–0.4 g/kg for protein [6,7,9]. Lastly, although the impact of the consumption of protein amino acids in handball players such as glutamine or branched amino acids has been studied [52], in our sample of athletes their consumption did not stand out, with both SS having less supporting scientific evidence according to the AIS and the current stances of prominent scientific organizations [6,7,9].

Regarding the analysis of SS consumption as a function of gender, for total group A supplements and performance supplements, it was observed that men consumed more of them, while women consumed more medical supplements. On the one hand, men tend to have a higher basal metabolic rate and greater muscle mass than women, which can lead to a greater need for performance-enhancing supplements such as protein powder and creatine to support muscle growth and recovery [53]. On the other hand, nutritional needs may vary by gender, with women often requiring higher intakes of micronutrients such as iron and calcium due to reproductive health issues, which may influence the choice of medical supplements [54]. The results of our research are similar in some respects to those found by Günalan et al. [17], who analyzed the consumption of soccer players and found that men consumed more performance supplements than women, but also consumed more medical supplements than women players. As for the differences according to the competitive level, for the consumption of the total supplements from group A and sports food, the players of the provincial level consumed the least, while for the performance supplements and the supplements from group B and C, the consumption increased as the level increased, with the players in the honor division consuming the most. Regarding the study by Günalan et al. [17], which compared professional and non-professional soccer players, it was also observed that professional players consumed more sports foods, performance supplements, group A total supplements, medical supplements and group C total supplements. These data are similar to those found in our study, considering that the honor division category was composed of professional handball players. This may be because when there is an increase in level, training days and/or physical demands, the consumption of supplements to improve performance and recovery may also increase [53]. In addition, it should also be considered that the consumption of SS in handball can vary depending on the budget allocation of the clubs. In general, it is common to observe that men’s teams, and especially those competing at higher levels, have a more substantial budget compared to semi-professional or women’s teams. In addition, the inclusion of a sports nutritionist can influence decision-making regarding supplementation, which is a beneficial factor, as he or she can recommend evidence-based supplements, considering the level of competition, gender, age and supplementation budget. Given the above, and with respect to the second hypothesis of this study, on the one hand, it is true that significant differences between men and women were not found for the total consumption of supplements, although significant differences were found when analyzing what type of supplements were consumed. On the other hand, for the competitive level, significant differences were found between total consumption, even observing that for some supplements the consumption increased as the level of competition increased. Although this was not observed for all supplements, it was observed that the provincial level, the lowest competitive level, was in all cases the one with the lowest SS consumption.

The last objective of this research was to verify if the SS consumed by handball players were supported by scientific evidence and to which group they belonged according to the AIS classification [10]. According to our results, the most consumed dietary supplements were mainly from the AIS category A, which has the highest scientific support. In relation to the consumption of prohibited substances (classified by the AIS in group D), the study sample did not show consumption of such products. This was to be expected as their use not only compromises sporting integrity, but also carries severe penalties and legal consequences that can jeopardize careers and reputations in the game [10,55]. In addition, in a previous study with a sample of handball players, it was observed that this type of athlete was not in the habit of using them [56]. Therefore, the last hypothesis of this research could be accepted. Nevertheless, there is still a lack of research on all these aspects of SS consumption, both in handball and in most sports.

### 4.1. Limitations

The present study encompasses certain limitations that necessitate consideration to enhance its applicability. While the deployment of the CSS questionnaire proved to be efficient in elucidating the behavioral patterns of the sampled population, it bears intrinsic limitations in the precise quantification of supplement usage, both in the context of training and competitive events. In addition, the questionnaire does not consider the doses or protocols of the SS consumed, nor does it ask about the brand of supplements. Therefore, it is not possible to estimate the energy and nutritional intake derived from SS. This limitation is present in all questionnaires regarding SS consumption frequency [57]. Another limitation was that the sample composition was heterogeneous, notably between the distinct competitive cohorts and across genders. Despite these limitations, the main objective of the study, which was to assess dietary trends and practices of SS consumption in handball athletes, was successfully accomplished. Notably, handball athletes may exhibit shifts in their supplementation habits over time, owing to alterations in nutritional requirements, evolving professional recommendations, or the influence of dietary supplement trends. The questionnaire employed in the study may not comprehensively capture such dynamic variations and might require periodic updates to accurately reflect alterations in consumption trends. Lastly, the survey instrument used to assess dietary supplement consumption was a validated tool, albeit it was constrained by its reliance on self-reported retrospective data collection found in the respondents’ memory.

### 4.2. Practical Applications

**Figure 1:**
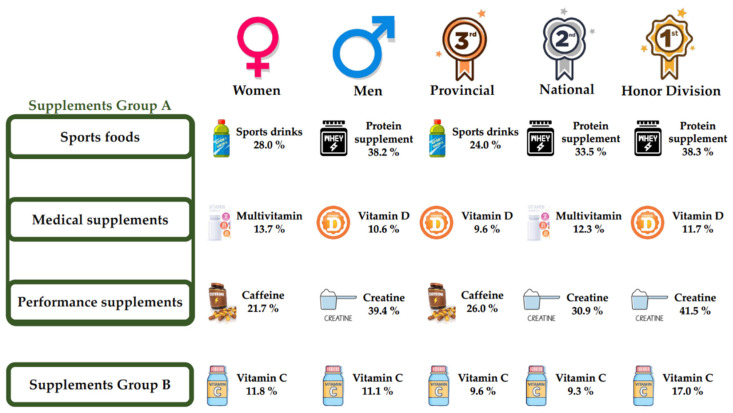
Most consumed supplements according to gender and competitive level, and in accordance with the categories established by the AIS [10].

The use of SS questionnaires in handball clubs is a useful means of obtaining data on the typology and quantities of SS ingested by handball athletes (Figure 1). In addition, it facilitates the evaluation of the effectiveness and perception of SS used by athletes. The use of this type of questionnaires on supplement consumption by the entire technical-medical staff around the players can also provide information on aspects related to the performance and recovery of each player. In this way, teams can have protocols, based on scientific evidence, about which supplements are safe and influence the improvement of each player [30]. In turn, it has the potential to unveil risk practices associated with the use of SS in handball players, including suboptimal doses, doping substances and inadvisable SS combinations that may lead to interactions with other SS or medication, causing adverse effects. These revelations are of utmost importance to promote the responsible use of dietary supplements in handball clubs. In conclusion, the application of these questionnaires provides data conducive to scientific research in the field of sports nutrition. These data, in turn, play a key role in advancing our understanding of the impact of SS on the performance, health and general well-being of players. Consequently, this knowledge promises to improve recommendations and guidelines in handball.

## 5. Conclusions

The percentage of handball players using SS was high. In terms of gender, men consumed more performance supplements and women more medical supplements. While at the competitive level, players in the honor division and at the national level consumed more SS than those at the provincial level, even performance supplements, group B supplements and group C supplements, players in the honor division had a higher consumption than those at the national and provincial levels. The SS most consumed by handball players were whey protein, sports drinks and creatine monohydrate, the latter being the most related to both gender and competitive level. Finally, and considering the AIS classification, the players did not consume any supplement from group D (banned or at high risk of contamination with substances that could lead to a positive doping test), with most of the choices being supplements from group A, which have the highest possible scientific evidence at present. This study includes relevant information for handball coaches and players about the use of SS that could enhance performance and recovery in handball, as well as the scientific evidence on the usefulness of these SS.

## Figures and Tables

**Table 1:** Descriptive data of the participants, according to gender and level of competition.

Variables	Gender (Mean ± SD)					Level of Competition (Mean ± SD)					
	Women (n = 161)	Men (n = 199)	t	*p*	d	Provincial (n = 104)	National (n = 162)	Honor Division (n = 94)	F	*p*	η^2^
Age (years)	22.53 ± 4.42	23.60 ± 5.99	−1.947	0.052	−0.200	23.19 ± 6.56	22.67 ± 4.66	23.82 ± 5.02	1.307	0.272	0.007
Body mass (kg)	66.25 ± 10.29	83.51 ± 12.51	−14.363	<0.001	−1.492	73.44 ± 13.61	78.25 ± 15.43	74.16 ± 12.77	4.486	0.012	0.025
Height (cm)	168.37 ± 5.84	181.65 ± 6.97	−19.659	<0.001	−2.046	174.07 ± 8.55	176.95 ± 9.23	175.39 ± 9.83	3.186	0.043	0.018
BMI * (kg/m^2^)	23.36 ± 3.43	25.24 ± 2.94	−5.602	<0.001	−0.594	24.11 ± 3.27	24.84 ± 3.69	23.96 ± 2.45	2.766	0.064	0.015
Training days	4.32 ± 1.21	3.99 ± 1.10	2.706	0.007	0.290	3.49 ± 0.91	3.91 ± 1.01	5.24 ± 0.84	92.973	<0.001	0.344
Years of experience	8.73 ± 2.14	8.67 ± 2.24	0.277	0.782	0.029	7.79 ± 2.90	8.93 ± 1.78	9.31 ± 1.54	14.405	<0.001	0.075

SD: standard deviation; t: Student’s *t* test value; d: Cohen’s d coefficient value; F: ANOVA analysis value; η^2^: eta squared value; BMI: body mass index; *: calculated from reported data on body mass and height.

**Table 2:** Descriptive data, Student’s *t* test and ANOVA of the sports supplements consumed in the different categories established by the AIS [10], according to gender and level of competition.

Variables		Gender (Mean ± SD)					Level of Competition (Mean ± SD)					
		Women	Men	t	*p*	d	P	N	HD	F	*p*	η^2^
Total SS		3.37 ± 3.64	3.81 ± 3.84	−1.125	0.261	−0.119	2.45 ± 2.94	3.90 ± 4.05	4.41 ± 3.75	7.869	<0.001	0.043
Group A	Total	2.24 ± 2.20	2.81 ± 2.55	−2.226	0.027	−0.236	1.82 ± 2.07	2.79 ± 2.67	2.97 ± 2.14	7.234	0.001	0.039
	Sport Food	1.39 ± 1.58	1.72 ± 1.85	−1.811	0.071	−0.192	1.11 ± 1.58	1.74 ± 1.91	1.79 ± 1.51	5.323	0.005	0.029
	Medical Supplements	0.42 ± 0.69	0.27 ± 0.61	2.240	0.024	0.241	0.26 ± 0.57	0.35 ± 0.68	0.39 ± 0.68	1.132	0.324	0.007
	Performance Supplements	0.43 ± 0.71	0.82 ± 0.88	−4.638	<0.001	−0.481	0.45 ± 0.69	0.70 ± 0.88	0.79 ± 0.85	4.591	0.011	0.024
Group B		0.33 ± 0.62	0.28 ± 0.64	0.716	0.474	0.076	0.17 ± 0.47	0.31 ± 0.62	0.43 ± 0.75	4.101	0.017	0.024
Group C		0.80 ± 1.41	0.72 ± 1.39	0.482	0.630	0.051	0.46 ± 0.93	0.79 ± 1.53	1.02 ± 1.52	4.125	0.017	0.022
Group D		0.00 ± 0.00	0.00 ± 0.00	0.000	1.000	0.000	0.00 ± 0.00	0.00 ± 0.00	0.00 ± 0.00	0.000	1.000	0.000

SD: standard deviation; t: Student’s *t* test value; d: Cohen’s d coefficient value; F: ANOVA analysis value; η^2^: eta squared value; SS: sports supplements; P: provincial; N: national; HD: honor division.

**Table 3:** Post hoc comparison between variables with significant differences according to the level of competition and according to the categories established by the AIS [10].

Variables		Group Comparison	Mean Difference ± SD	*p*	CI 95%
Total SS		P–N	−1.44 ± 0.46	0.006	−2.556 to −0.330
		P–HD	−1.96 ± 0.52	0.001	−3.224 to −0.702
		N–HD	−0.52 ± 0.48	0.831	−1.669 to 0.629
Group A	Total	P–N	−0.97 ± 0.30	0.004	−1.691 to −0.255
		P–HD	−1.15 ± 0.34	0.002	−1.964 to −0.338
		N–HD	−0.18 ± 0.31	1.000	−0.919 to 0.563
	Sport Food	P–N	−0.63 ± 0.22	0.011	−1.155 to −0.115
		P–HD	−0.68 ± 0.24	0.017	−1.271 to −0.092
		N–HD	−0.05 ± 0.22	1.000	−0.584 to 0.491
	Performance Supplements	P–N	−0.25 ± 0.10	0.054	−0.494 to 0.003
		P–HD	−0.33 ± 0.12	0.013	−0.617 to −0.054
		N–HD	−0.09 ± 0.11	1.000	−0.346 to 0.167
Group B		P–N	−0.14 ± 0.08	0.214	−0.330 to 0.047
		P–HD	−0.25 ± 0.09	0.014	−0.466 to −0.039
		N–HD	−0.11 ± 0.08	0.515	−0.305 to 0.084
Group C		P–N	−0.33 ± 0.17	0.179	−0.747 to 0.090
		P–HD	−0.56 ± 0.20	0.014	−1.034 to −0.086
		N–HD	−0.23 ± 0.18	0.596	−0.663 to 0.201

SD: standard deviation; CI: confidence interval; SS: sports supplements; P: provincial; N: national; HD: honor division.

**Table 4:** Distribution (%) of the most consumed supplements according to gender and level of competition, and according to the categories established by the AIS [10].

Category		Supplement	Total (%)	Gender (%)							Level of Competition (%)								
				Women	Residual	Men	Residual	X^2^	*p*	V	P	Residual	N	Residual	HD	Residual	X^2^	*p*	V
Supplements Group A	Sport Food	Sports Drinks	30.8	28.0	−1.1	33.2	1.1	1.135	0.287	0.056	24.0	−1.8	32.7	0.7	35.1	1.0	3.326	0.190	0.096
		Sports Bars	27.0	27.3	0.1	26.8	−0.1	0.014	0.905	0.006	20.2	−1.9	29.6	1.0	30.1	0.8	3.468	0.177	0.098
		Protein Bars	25.6	25.5	0.0	25.6	0.0	0.001	0.972	0.002	16.0	−2.6	29.0	1.4	29.8	1.1	6.539	0.038	0.135
		Whey Protein	30.4	20.8	−3.6	38.2	3.6	12.689	<0.001	0.188	18.4	−3.1	33.5	1.1	38.3	1.9	10.468	0.005	0.171
	Medical Supplements	Vitamin D	10.6	10.6	0.0	10.6	0.0	0.000	0.998	0.000	9.6	−0.4	10.5	0.0	11.7	0.4	0.229	0.892	0.025
		Multivitamin	10.8	13.7	1.6	8.5	1.6	2.417	0.120	0.082	8.7	−0.8	12.3	0.8	10.6	−0.1	0.899	0.638	0.050
	Performance Supplements	Caffeine	28.6	21.7	−2.6	34.2	2.6	6.734	0.009	0.137	26.0	−0.7	30.2	0.6	28.7	0.0	0.570	0.752	0.040
		Creatine Monohydrate	29.2	16.8	−4.7	39.4	4.7	21.963	<0.001	0.247	15.5	−3.6	30.9	0.6	41.5	3.0	16.373	<0.001	0.214
Supplements Group B		Antioxidant Vitamin C	11.4	11.8	0.2	11.1	−0.2	0.049	0.825	0.012	9.6	−0.7	9.3	−1.2	17.0	2.0	4.007	0.135	0.106

X^2^: chi-square test value; V: Cramer’s V value; P: provincial; N: national; HD: honor division.

## Data Availability

The data presented in this study are available in the tables of this article. The database of this study can be obtained from the corresponding author.

## Supplementary Materials

The following supporting information can be downloaded at https://www.mdpi.com/article/10.3390/nu16020315/s1.

## Abbreviations

SS: sport supplements; AIS: Australian Institute of Sport; SD: standard deviation; BMI: body mass index; P: provincial; N: national; HD: honor division; CI: confidence interval; CSS: consumption of sports supplements. RFEBM: Spanish Handball Federation.
