# Supplementary material for: Pattern of Consumption of Sports Supplements of Spanish Handball Players: Differences According to Gender and Competitive Level
Source: Nutrients. 2024 Jan 20;16(2):315. doi: 10.3390/nu16020315 (PMC10820419; doi:10.3390/nu16020315)
Supplement: Supplementary file 1 [file nutrients-16-00315-s001.zip › nutrients-2753256-supplementary.pdf]

Balonmano - Cuestionario Consumo de Suplementos Nutricionales y Alimentos

Título investigación: Estudio del consumo de alimentos y suplementos nutricionales en balonmano.

El objetivo de este estudio es determinar y comparar con la evidencia científica actual el consumo de suplementos nutricionales y alimentos de jugadores/as de balonmano.

Se trata de un cuestionario CONFIDENCIAL. Toda la información será analizada por personal técnico sujeto al secreto profesional y utilizada exclusivamente para los fines descritos. La Ley Orgánica 3/2018 de Protección de Datos será respetada en todo momento. Este estudio ha sido aprobado por el comité de ética de la Universidad de Alicante (UA-2022-02-01) y cuenta con la colaboración de la Real Federación Española de Balonmano (RFEBM). En el informe de resultados no podrán ser identificadas las respuestas de ninguna persona de forma individualizada.

Para participar en el estudio es necesario ser mayor de edad y contestar INDIVIDUALMENTE. Por ello te pedimos que respondas siguiendo las instrucciones de cada pregunta para contestar. Es mu importante que contestes TODAS LAS PREGUNTAS ya que muchas se tratan conjuntamente, de manera que si dejas alguna pregunta sin contestar es probable que otras que hayas contestado se anulen y se pierda la información para el análisis de resultados.

Para llevar a cabo esta investigación se ha creado un Grupo de Trabajo formado: David Romero (drg33@gcloud.ua.es), Raquel Vaquero (rvaquero@ucam.edu) y José Miguel Martínez (josemiguel.ms@ua.es). Si necesitas realizar alguna consulta te puedes dirigir a cualquiera de sus miembros.

MUCHAS GRACIAS POR TU COLABORACIÓN

CONSENTIMIENTO:
- Soy mayor de edad.
- He sido informado/a de las características del Proyecto de Investigación titulado: “Estudio del consumo de alimentos y suplementos nutricionales en balonmano”.
- He leído la información del proyecto, y he podido formular las dudas que me han surgido al respecto.
- Considero que he entendido dicha información.
- Estoy informado/a de la posibilidad de retirarme en cualquier momento del estudio.
- Estoy informado del modo en que serán tratados mis datos.
- No rellenar más de una inscripción por persona y escribir en MAYÚSCULAS todas las respuestas que requieran texto

* Indica que la pregunta es obligatoria

1. En virtud de tales condiciones ¿consientes participar en este estudio? *

   Marca solo un óvalo.

   - Si
   - No

Parte 1. DATOS INICIALES

Sección compuesta por 6 preguntas.

2. 1. SEXO *

   Marca solo un óvalo.

   - Hombre
   - Mujer
   - No sabe / no contesta

3. 2. FECHA DE NACIMIENTO *

   Ejemplo: 7 de enero del 2019

4. 3. EDAD ACTUAL EN AÑOS *

   Indicar edad en números

5. 4. COMUNIDAD AUTÓNOMA DE RESIDENCIA *

Marca solo un óvalo.

- Andalucía
- Cataluña
- Comunidad de Madrid
- Comunidad Valenciana
- Galicia
- Castilla y León
- País Vasco
- Canarias
- Castilla-La Mancha
- Región de Murcia
- Aragón
- Isla Baleares
- Extremadura
- Principado de Asturias
- Navarra
- Cantabria
- La Rioja
- Melilla
- Ceuta

6. 5. ALTURA APROXIMADA EN CENTÍMETROS (cm) *

En centímetros

7. 6. PESO APROXIMADO EN KILOGRAMOS (kg) *

En kilogramos

Parte 2. PRÁCTICA DEL DEPORTE

Sección compuesta por 6 preguntas.

8. 1. ¿ESTAS FEDERADO? *

Marca solo un óvalo.

- Sí
- No

9. 2. INDICA EL NÚMERO DE AÑOS APROXIMADO QUE LLEVAS FEDERADO *

Marca solo un óvalo.

- 1 año
- 2 años
- 3 años
- 4 años
- 5 años
- 6 años
- 7 años
- 8 años
- 9 años
- 10 o más años

10. 3. INDICA EN EL NIVEL QUE COMPITES ACTUALMENTE *

Señala el de mayor categoría

Marca solo un óvalo.

- Provincial/Territorial
- Nacional
- División de Honor

11. 4. INDICA EN QUE LIGA ESTÁS COMPITIENDO *

Marca solo un óvalo.

- División de Honor
- División de Honor Plata
- 1º Nacional
- 2º Nacional
- Territorial

12. 5. INDICA LOS AÑOS QUE LLEVAS COMPITIENDO EN EL NIVEL SEÑALADO ANTERIORMENTE

Marca solo un óvalo.

- 1 año
- 2 años
- 3 años
- 4 años
- 5 años
- 6 años
- 7 años
- 8 años
- 9 años
- 10 o más años

13. 6. INDICA SI PERTENECES ALGÚN PROGRAMA DE TECNIFICACIÓN *

Marca solo un óvalo.

- Si
- No

Parte 2. ENTRENAMIENTO Y COMPETICIÓN

Sección compuesta por 4 preguntas.

14. 1. ¿CUÁNTOS DÍAS DE MEDIA DEDICAS AL ENTRENAMIENTO A LA SEMANA? *
APROXIMADAMENTE

Marca solo un óvalo.

- 1 día
- 2 días
- 3 días
- 4 días
- 5 días
- 6 días
- 7 días

15. 2. ¿CUÁNTO TIEMPO, APROXIMADAMENTE, DEDICAS CADA DÍA AL ENTRENAMIENTO?
Elige el intervalo de tiempo que más se ajuste a tu tiempo de entrenamiento

Marca solo un óvalo.

- <30 minutos
- Entre 30 minutos y 1 hora
- Entre 1 hora y 1,5 horas
- Entre 1,5 horas y 2 horas
- >2 horas

16. 3. SUELES ENTRENAR... *

Elige el intervalo de tiempo que más se ajuste a tu tiempo de entrenamiento

Marca solo un óvalo.

- Por la mañana
- Por la tarde
- Por la mañana y la tarde
- Otro: ____________________

17. 4. LOS PARTIDOS SUELEN SER... *

Elige el intervalo de tiempo que más se ajuste al momento del partido

Marca solo un óvalo.

- Por la mañana
- Por la tarde
- Por la mañana y la tarde
- Otro: ____________________

Parte 3. SUPLEMENTOS NUTRICIONALES

18. ¿ESTÁS A FAVOR DEL CONSUMO DE SUPLEMENTOS EN LA ACTIVIDAD FÍSICA DENTRO DE LA LEGALIDAD?

Marca solo un óvalo.

- Si
- No
- No sabes/No contestas

19. ¿HAS CONSUMIDO EN ALGUNA OCASIÓN ALGÚN SUPLEMENTO NUTRICIONAL? *

Marca solo un óvalo.

- Si *Salta a la pregunta 20*
- No

SUPLEMENTOS CONTINUACIÓN

20. SEÑALA CUALES DE ESTOS SUPLEMENTOS CONSUMES DE MANERA HABITUAL *

ESTÁN ORDENADOS ALFABÉTICAMENTE. PUEDES SEÑALAR TANTOS CUANTOS CONSUMAS D MANERA HABITUAL

Selecciona todos los que correspondan.

- [ ] 5-HTP (5-Hidroxitriptofano)
- [ ] ATP
- [ ] Ácido Aspártico
- [ ] Ácido Alfa Lipoico (ALA)
- [ ] Ácido Fosfatídico
- [ ] Ácido Hialurónico
- [ ] Ácidos Grasos ω-3
- [ ] Ácidos Grasos ω-6
- [ ] Ácidos Grasos ω-9
- [ ] Ácido Linoleico Conjugado (CLA)
- [ ] Aceite de Coco
- [ ] Aceite de Hígado de Bacalao
- [ ] Aceite de Lino
- [ ] Aceite de Onagra
- [ ] Aceite de Prímula
- [ ] Amilopectina
- [ ] Aminoácidos esenciales (EEAA)
- [ ] Aminoácidos ramificados (BCAA)
- [ ] Arginina
- [ ] Ashwagandha
- [ ] Barritas Energéticas o Deportivas
- [ ] Barritas Proteicas
- [ ] Bebida Isotónica o de Reposición
- [ ] Beta Alanina

- [ ] Bicarbonatos
- [ ] Bloqueadores de Carbohidratos
- [ ] Cafeína (capsula, pastilla, liquido o chicle)
- [ ] Carbohidratos ("Gainers")
- [ ] Carnitina (Acetil-L-Carnitina)
- [ ] Carnitina (L-Carnitina)
- [ ] Cartílago de Tiburón
- [ ] Caseína Micelar
- [ ] Chitosan
- [ ] Ciclodextrinas
- [ ] Cinc
- [ ] Citrulina (Malato o L-citrulina)
- [ ] Complejo Mineral
- [ ] Complejo Vitamínico
- [ ] Colágeno
- [ ] Condroitina
- [ ] Creatina (Monohidrato)
- [ ] Creatina (Kre-Alkalyn)
- [ ] Creatina (Etil Éster)
- [ ] Curcumina
- [ ] Dextrosa
- [ ] Dimetilglicina
- [ ] Diuréticos
- [ ] Electrolitos (en polvo o pastillas)
- [ ] Epicatequina
- [ ] Epigalocatequina-3-galato (EGCG)
- [ ] Espirulina
- [ ] Fórmulas pre-entreno
- [ ] Fosfato de sodio
- [ ] Geles deportivos
- [ ] Ginseng
- [ ] Glicerol
- [ ] Glucosamina
- [ ] Glutamina
- [ ] Gominolas
- [ ] Greens
- [ ] Guarana
- [ ] Hidrolizado de caseína
- [ ] Hidroximetilbutirato (HMB)
- [ ] Hierro

- [ ] Isomaltulosa
- [ ] Jalea Real
- [ ] Lecitina de Soja
- [ ] Leucina
- [ ] Levadura de Cerveza
- [ ] Magnesio
- [ ] Maltodextrina
- [ ] Melatonina
- [ ] Mentol
- [ ] Metil-sulfonil-metano (MSM)
- [ ] Nitrato (zumo de remolacha)
- [ ] Nootrópicos
- [ ] N-Acetilcisteína
- [ ] Picolinato de Cromo
- [ ] Polen
- [ ] Potenciador de Testosterona
- [ ] Precursor Hormonal
- [ ] Probióticos
- [ ] Proteína de Carne
- [ ] Proteína de Suero (Whey Protein)
- [ ] Proteína Vegetal (soja, cáñamo…)
- [ ] Quinina
- [ ] Quercetina
- [ ] Recuperadores (mezcla de carbohidratos y proteínas)
- [ ] Ribosa
- [ ] Sinefrina (p-sinefrina)
- [ ] Suplemento de cereza ácida
- [ ] Suplemento de cetonas
- [ ] Taurina
- [ ] Teanina
- [ ] Teacrina
- [ ] Té verde (completo o extractos)
- [ ] Tribulus
- [ ] Tirosina
- [ ] Triglicéridos de cadena media (MCT)
- [ ] Vitamina C
- [ ] Vitamina D
- [ ] Vitamina E
- [ ] Vitamina K
- [ ] Yohimbina

- [ ] Zinc
- [ ] ZMA
- [ ] Otro: ____________________

21. INDICA QUÉ DÍAS DE PRACTICA DEPORTIVA SUELES CONSUMIRLOS *

Marca solo un óvalo.

- ( ) Entrenamiento *Salta a la pregunta 20*
- ( ) Competición
- ( ) Entrenamiento y competición
- ( ) Periodo vacacional o de descanso
- ( ) En todos lo casos anteriormente mencionados
- ( ) Otro: ____________________

22. INDICA CUANDO SUELES CONSUMIRLOS *

Selecciona todos los que correspondan.

- [ ] Antes de la práctica deportiva
- [ ] Durante la práctica deportiva
- [ ] Después de la práctica deportiva
- [ ] En todos los casos anteriores
- [ ] Indiferentemente
- [ ] Otro: ____________________

23. ¿CON QUÉ FIN LOS CONSUMES? *

Señala la más frecuente

Marca solo un óvalo.

- Para cuidar tu salud
- Por problemas de salud
- Por necesidad
- Por obligación
- Para mejorar su aspecto físico
- Para buscar rendimiento deportivo
- Por paliar algún déficit de la dieta
- Otro: ____________________

24. ¿DONDE SUELES COMPRAR LOS SUPLEMENTOS? *

Señala la más frecuente

Marca solo un óvalo.

- En una Farmacia
- En un Gimnasio
- A un Amigo
- A un Monitor
- A un Dietista-Nutricionista
- En un Herbolario
- En un Centro Comercial
- En una Parafarmacia
- En Internet
- En una Tienda especializada
- Otro: ____________________

25. ¿QUÉ/QUIEN TE MOTIVÓ A TOMARLOS? *

Señala la más frecuente

Marca solo un óvalo.

- Compañero de equipo
- Entrenador
- Internet
- Monitor
- Publicidad
- Dietista-Nutricionista
- Revista especializada
- Televisión
- Preparador Físico
- Médico
- Amigo
- Otro: ____________

26. EN GENERAL, ¿CREES QUE TE DIERON RESULTADOS? *

Marca solo un óvalo.

	1	2	3	4	5	
NIN	◯	◯	◯	◯	◯	MUCHO RESULTADO

27. SI FUERA EL CASO, ESCRIBE ALGÚN/OS DE LOS SUPLEMENTOS QUE CREES QUE NO TE DIERON NINGÚN RESULTADO

____

28. SI FUERA EL CASO, ESCRIBE ALGÚN/OS DE LOS SUPLEMENTOS QUE CREES QUE SI TE DIERON RESULTADO

29. ¿HAS CONSUMIDO O CONSUMIRÍAS ALGÚN SUPLEMENTO QUE FUERA PERJUDICIAL PARA LA SALUD PERO QUE TE AYUDARA A CONSEGUIR TUS OBJETIVOS?

Marca solo un óvalo.

- Si
- No
- No sabe/No contesta

30. EN EL CASO QUE LA PREGUNTA ANTERIOR SEA AFIRMATIVA, SEÑALA CUÁL/ES DE LA SIGUIENTE LISTA HAS CONSUMIDO O CONSUMIRÍAS

Selecciona todos los que correspondan.

- [ ] Esteroides anabolizantes
- [ ] Hormona de crecimiento
- [ ] Insulina
- [ ] Efedrina
- [ ] Pseudoanfetaminas
- [ ] Otro: ____________________

31. INDICA, DESDE TU PUNTO DE VISTA, EL USO DE ESTE TIPO DE SUSTANCIAS PERJUDICIALES EN TU DEPORTE

Marca solo un óvalo.

	1	2	3	4	5	
NIN	◯	◯	◯	◯	◯	MUCHO USO

Parte 4. CONSUMO DE ALIMENTOS Y DIETA

Sección sobre consumo de alimentos conformada por 18 preguntas.

32. 1. ¿Cuántas piezas de fruta has consumido en los últimos 28 días?(1 pieza = aproximadamente 100 g) (incluido zumos de fruta) (1 porción = Aprox. 200 ml)

Marca solo un óvalo.

- ◯ Menos de una vez a la semana
- ◯ Una o dos veces a la semana
- ◯ 3-4 veces a la semana
- ◯ 5-6 Veces a la semana
- ◯ Una o dos veces al día
- ◯ 3 veces o más al día

33. 2. ¿Cuántas porciones de verduras has consumido en los últimos 28 días? (1 porción = aproximadamente 150 g)

Marca solo un óvalo.

- Menos de una vez a la semana
- Una o dos veces a la semana
- 3-4 veces a la semana
- 5-6 Veces a la semana
- Una o dos veces al día
- 3 veces o más al día

34. 3. ¿Cuántas porciones de legumbres (lentejas, garbanzos, judías blancas, etc.) has consumido en los ultimos 28 dias? (1 porción = aprox. 60 g)

Marca solo un óvalo.

- Menos de una vez a la semana
- Una o dos veces a la semana
- 3-4 veces a la semana
- 5-6 Veces a la semana
- Una o dos veces al día
- 3 veces o más al día

35. 4. ¿Cuántas porciones de carne blanca (pollo / pavo) has consumido en los últimos 28 días? (1 porción = aproximadamente 150 g)

Marca solo un óvalo.

- Menos de una vez a la semana
- Una o dos veces a la semana
- 3-4 veces a la semana
- 5-6 Veces a la semana
- Una o dos veces al día
- 3 veces o más al día

36. 5. ¿Cuántas porciones de pescado has consumido en los últimos 28 días (1 porción = aproximadamente 150 g)

Marca solo un óvalo.

- Menos de una vez a la semana
- Una o dos veces a la semana
- 3-4 veces a la semana
- 5-6 Veces a la semana
- Una o dos veces al día
- 3 veces o más al día

37. 6. ¿Cuántas porciones de carne roja (carne, cerdo, cordero) has consumido en los últimos 28 días? (1 porción = aproximadamente 150 g)

Marca solo un óvalo.

- Menos de una vez a la semana
- Una o dos veces a la semana
- 3-4 veces a la semana
- 5-6 Veces a la semana
- Una o dos veces al día
- 3 veces o más al día

38. 7. ¿Cuántas porciones de refrescos has consumido en los últimos 28 días? (1 porción = aproximadamente 250 ml)

Marca solo un óvalo.

- Menos de una vez a la semana
- Una o dos veces a la semana
- 3-4 veces a la semana
- 5-6 Veces a la semana
- Una o dos veces al día
- 3 veces o más al día

39. 8. ¿Cuántas porciones de dulces producidos comercialmente (no hechos en casa) (Galletas / pasteles) has consumido en los últimos 28 días? (1 pieza = aproximadamente 100 g)

Marca solo un óvalo.

- Menos de una vez a la semana
- Una o dos veces a la semana
- 3-4 veces a la semana
- 5-6 Veces a la semana
- Una o dos veces al día
- 3 veces o más al día

40. 9. ¿Cuántas porciones de alimentos preparados / congelados has consumido (Croquetas, pizza, etc.) en los últimos 28 días? (1 porción = Aprox. 80 g)

Marca solo un óvalo.

- Menos de una vez a la semana
- Una o dos veces a la semana
- 3-4 veces a la semana
- 5-6 Veces a la semana
- Una o dos veces al día
- 3 veces o más al día

41. 10. ¿Ha consumido bebidas alcohólicas en los últimos 28 días? *

Marca solo un óvalo.

- Sí
- No

42. 11. ¿Qué tipo de bebidas alcohólicas ha consumido en los últimos 28 días? *

Selecciona todos los que correspondan.

- [ ] Cerveza
- [ ] Vino
- [ ] Bebidas mezcladas
- [ ] No he consumido bebidas alcohólicas

43. 12. ¿Cuántas porciones de cerveza / vino / licores o bebidas mezcladas has consumido e los últimos 28 días? (1 porción de cerveza = aproximadamente 200 ml / 1 Vaso de vino = aprox. 100 ml / 1 porción de licores o bebidas mezcladas =Aprox. 50 ml (de alcohol)

Marca solo un óvalo.

- Menos de una vez a la semana
- Una o dos veces a la semana
- 3-4 veces a la semana
- 5-6 Veces a la semana
- Una o dos veces al día
- 3 veces o más al día

Parte 4. CONSUMO DE ALIMENTOS Y DIETA (continuación)

44. ¿SIGUES EN LA ACTUALIDAD ALGÚN TIPO DE DIETA O FORMA CONCRETA DE ALIMENTARSE?

Marca solo un óvalo.

- Si
- No

45. ¿QUÉ TIPO DE DIETA ES? *

Marca solo un óvalo.

- Vegetariana
- Vegana
- Mediterránea
- Ovolactovegetariana
- Paleo
- Disociada
- Macrobiotica
- Flexible
- Cetogénica
- No sigo dieta
- Otro: ______________________

46. ¿POR QUÉ LA HACES? *

Señala el motivo principal

Marca solo un óvalo.

- Para cuidar tu salud
- Por estética
- Por enfermedad
- Por rendimiento
- Por religión o creencias
- No sigo dieta
- Otro: ______________________

47. ¿TE LLEVA ALGUIEN O TE APOYAS EN ALGO PARA LLEVAR LA DIETA? *
EN CASO AFIRMATIVO INDICA LA RESPUESTA ADECUADA.

Marca solo un óvalo.

- No
- Dietista-Nutricionista
- Médico
- Farmaceutico
- Entrenado Personal
- Fisioterapeuta
- Alguien sin ningula titulación
- Me apoyo en un libro
- Me apoyo en un blog
- Me apoyo en un amigo
- Me apoyo en Redes Sociales
- No sigo dieta
- Otro: ______________________

PARTE 5. HIDRATACIÓN

En esta última sección os preguntamos por vuestro consumo de AGUA (únicamente agua, sin incluir bebidas de reposición, bebidas isotónicas, etc, que contienen hidratos de carbono o sales minerales).

48. ¿CUANTOS LITROS DE AGUA SUELES BEBER EN UN DÍA? *

Marca solo un óvalo.

- 0,5 L
- 1 L
- 1,5 L
- 2 L
- 2,5 L
- Más de 2,5 L

49. ¿CUANTOS LITROS DE AGUA SUELES BEBER DURANTE LOS ENTRENAMIENTOS?

Marca solo un óvalo.

- 0,5 L
- 1 L
- 1,5 L
- 2 L
- 2,5 L
- Más de 2,5 L
- No suelo beber agua

50. ¿CUANTOS LITROS DE AGUA SUELES BEBER DURANTE LOS PARTIDOS? *

Marca solo un óvalo.

- 0,5 L
- 1 L
- 1,5 L
- 2 L
- 2,5 L
- Más de 2,5 L
- No suelo beber agua

Este contenido no ha sido creado ni aprobado por Google.

Google Formularios
